# Two-Stage Mucogingival Surgery with Free Gingival Autograft and Biomend Membrane and Coronally Advanced Flap in Treatment of Class III Millers Recession

**DOI:** 10.1155/2016/9289634

**Published:** 2016-07-25

**Authors:** Avita Rath, Smrithi Varma, Renny Paul

**Affiliations:** ^1^Faculty of Dentistry, Segi University, 47810 Kota Damansara, Selangor, Malaysia; ^2^Department of Periodontics, Sri Sankara Dental College and Hospital, Varkala, Kerala 695318, India; ^3^Department of Periodontics, Al-Azhar Dental and Hospital, Perumpillichira, Thodupuzha, Kerala 685605, India

## Abstract

*Introduction*. Gingival recession is an apical shift of the gingival margin with exposure of the root surface. This migration of the marginal tissue leads to esthetic concerns, dentin hypersensitivity, root caries, and cervical wear. It is, paradoxically, a common finding in patients with a high standard of oral hygiene, as well as in periodontally untreated populations with poor oral hygiene. Changing the topography of the marginal soft tissue in order to facilitate plaque control is a common indication for root coverage procedures and forms a major aspect of periodontal plastic surgeries. The regeneration of a new connective tissue attachment to denuded root surface is by allowing the selective coronal regrowth of periodontal ligament cells while excluding the gingival tissues from the root during wound healing by means of a barrier membrane.* Case Presentation*. This case reports a two-stage surgical technique for treatment of Miller's class III defect using free gingival autograft and type I absorbable collagen membrane (BioMend®,* Zimmer Dental, USA*)^§^.* Conclusions*. The 6-month follow-up of the case showed a significant increase in attached gingiva suggesting it as a predictable alternative in the treatment of Millers class III defects.

## 1. Background

Successful coverage of exposed roots for esthetics and functional reasons has been the objective of various mucogingival surgeries. When adequate gingiva exists, repositioning it over the denuded root surface provides the most esthetic result [1]. However, this may not be seen in all the cases. Various factors need to be taken into consideration before deciding on the technique for root coverage [2]. Procedures are being constantly modified or used in combination to achieve successful and predictable root coverage [3, 4].

The aim of this case report is to demonstrate that a two-step surgical procedure using a free gingival graft and a GTR membrane is suitable and successful in areas that have a lack of attached gingiva and deep recession.

## 2. Case Presentation

**Figure 1:**
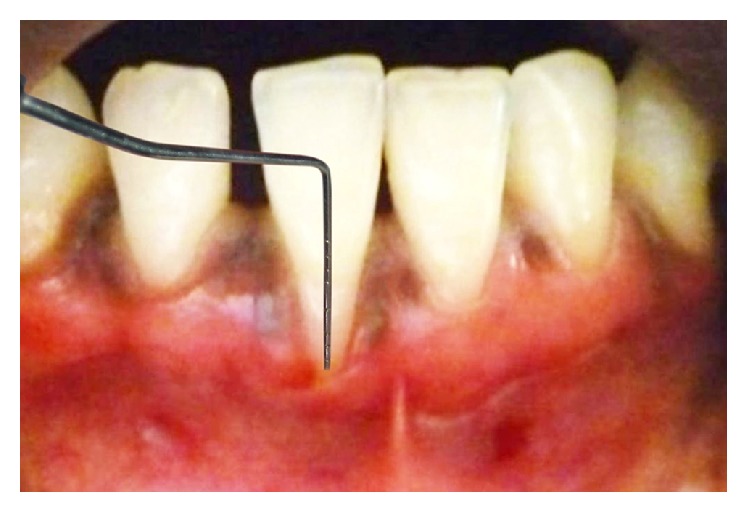
8 mm gingival recession at 41 region.

**Figure 2:**
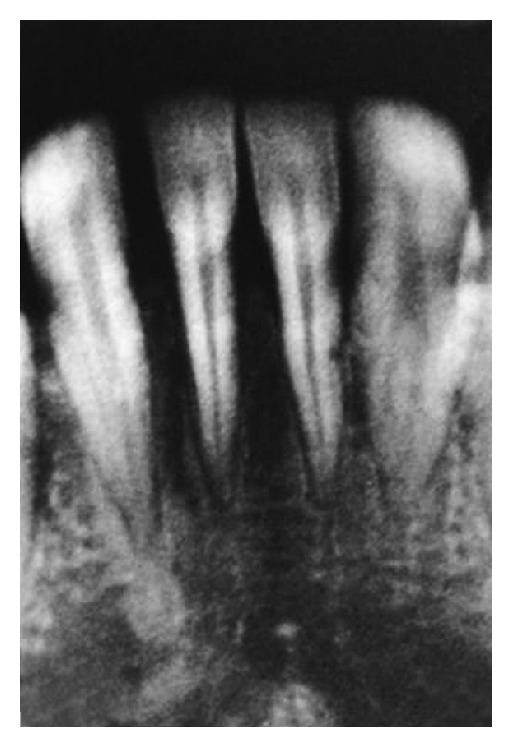
Preoperative radiograph of 41 region with interdental bone loss.

A 23-year-old male patient reported to the outpatient department, with chief complaint of receding gums and hypersensitivity in lower anterior region with difficulty in brushing in that region. He had a negative history of increasing recession and spacing between the teeth in the same or other regions and no familial history of the same, ruling out probability of aggressive periodontal disease. Moreover, he had no previous orthodontic treatment. His oral hygiene was good and there was no bleeding on probing. On examination the area of chief complaint revealed 8 mm of recession at 41 region with thin and narrow attached gingiva of 1 mm. Radiographic examination revealed interproximal bone loss diagnosing it to be a class III Millers recession [5] (Figures 1 and 2).

## 3. Case Management

Before starting the treatment, the treatment plan was thoroughly explained to the patient and a written consent was taken before initiation of the therapy.

Periodontal therapy included Phase 1 therapy of thorough scaling and root planning, initiated four weeks prior to the surgery [6].

### 3.1. Stage 1: Use of a Free Gingival Graft

**Figure 3:**
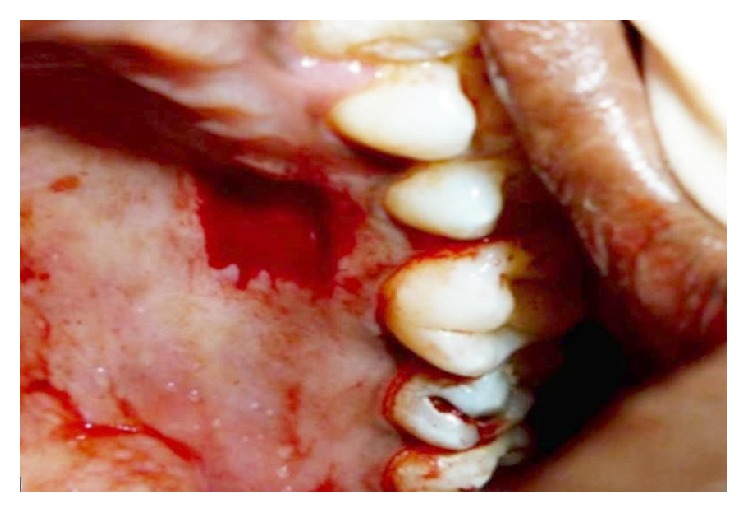
Palatal donor site.

**Figure 4:**
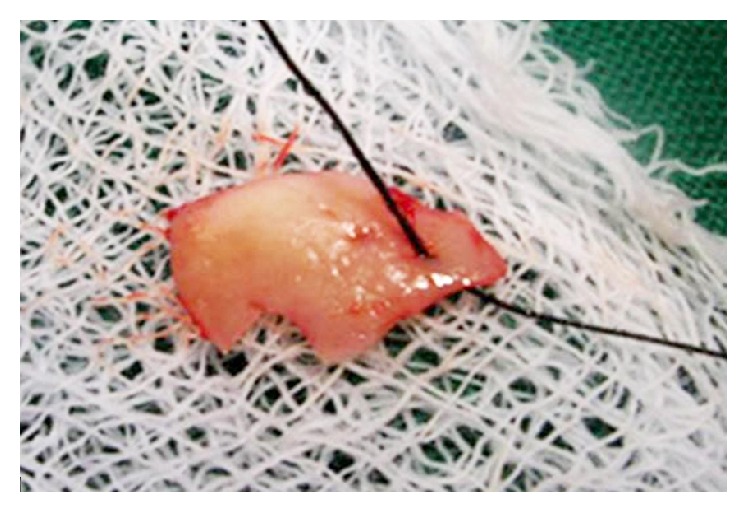
FGG harvested.

**Figure 5:**
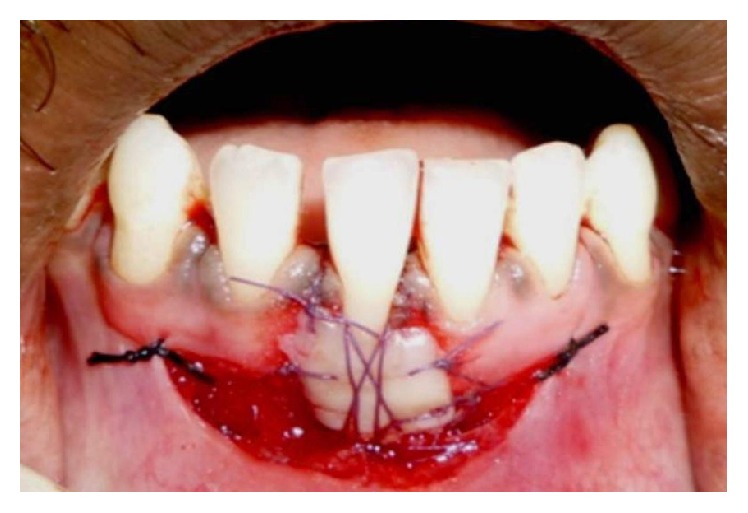
Graft stabilized with sutures.

The first step of the surgery used a free gingival graft (FGG) technique as given by Miller Jr. [7]. The recipient site was prepared under local anesthesia. The outline of the graft was obtained using a tin foil template with number 15 BP blade. The FGG was harvested from palate (Figures 3 and 4). The graft was adapted over the root and stabilized by horizontal and circumferential sutures using 4.0 vicryl sutures (Figure 5). The patient was recalled for review once every week for two months.

### 3.2. Stage II: Use of BioMend GTR Membrane

**Figure 6:**
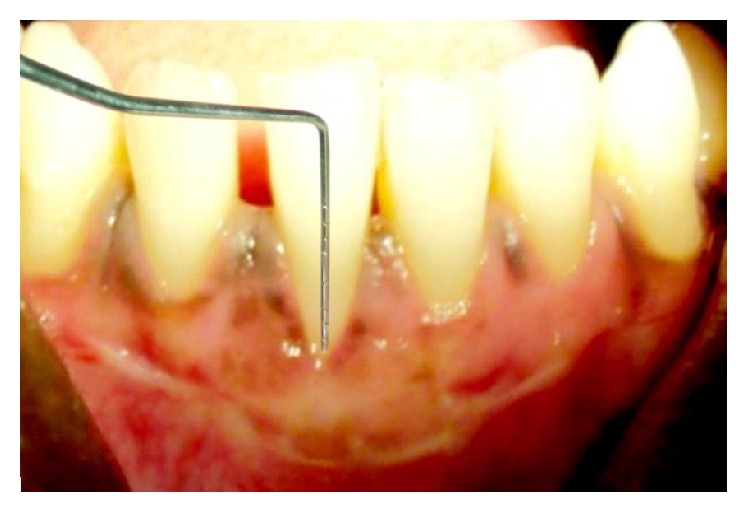
Postoperative view after 3 months.

**Figure 7:**
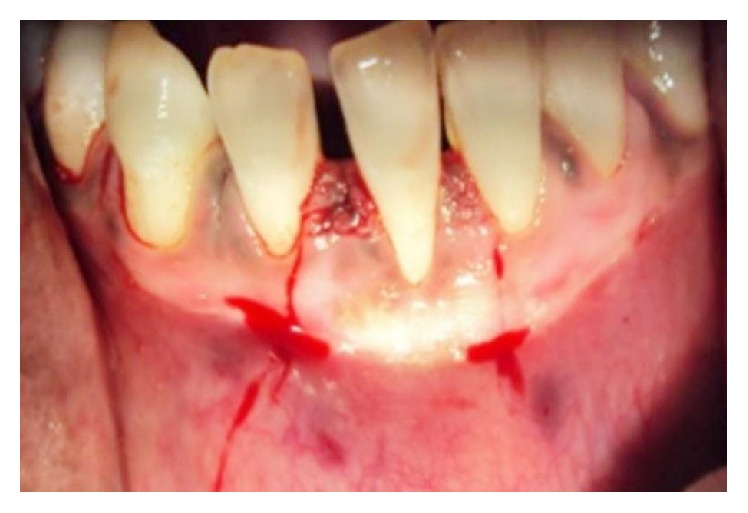
Vertical and interdental incisions made.

**Figure 8:**
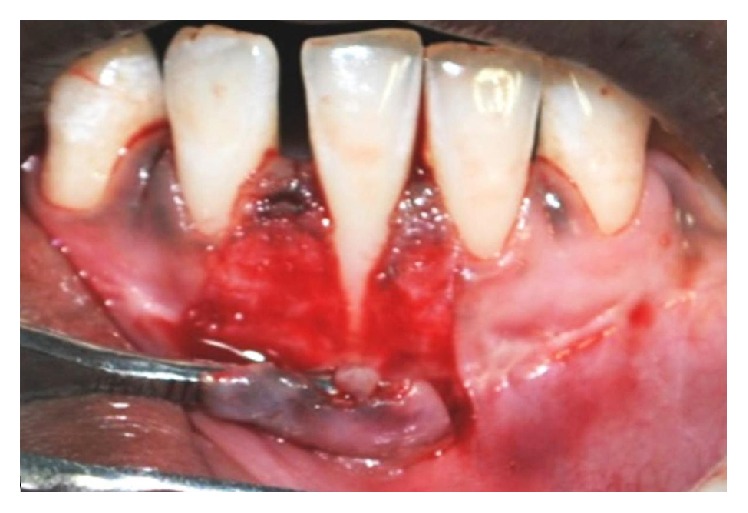
Partial thickness flap elevated.

**Figure 9:**
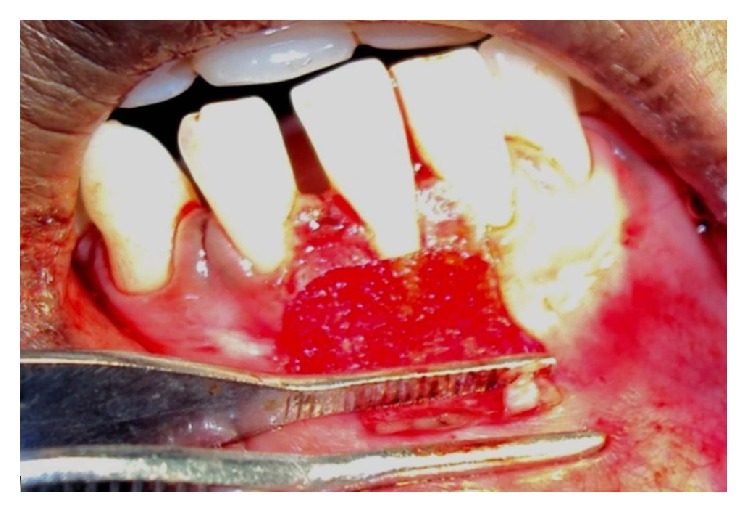
Biomend GTR membrane placed.

**Figure 10:**
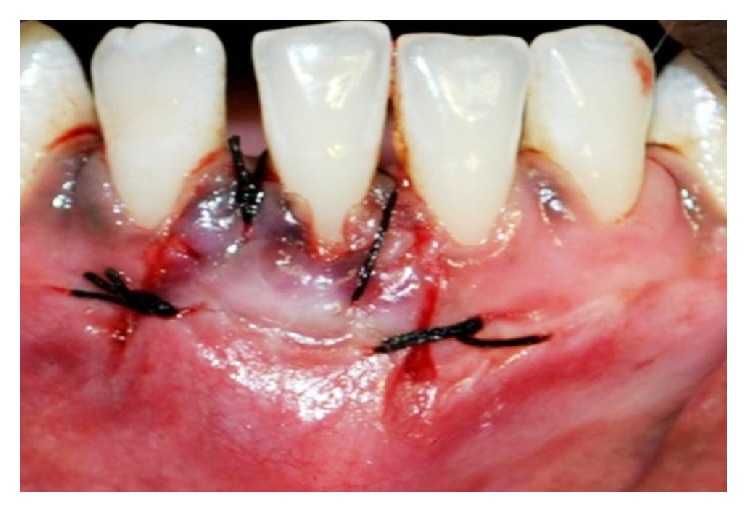
Flap coronally advanced and sutures placed.

The second stage of the surgery was performed 3 months after the first procedure (Figure 6). Using number 15 BP blade, a crevicular incision was given around 41 along with two oblique incisions (including the adjacent papillae) extending up to mucogingival junction (Figure 7). Partial thickness flap was elevated and the membrane (size 15 mm × 20 mm) was removed from its sterile pack and placed at the recipient site and adapted to the root surface (Figures 8 and 9). Flap was coronally repositioned and sutured using 4-0 sling vicryl sutures (Figure 10). The site was protected using a tin foil and periodontal dressing.

The patient was recalled once a month for the next six months.

## 4. Case Outcome

**Figure 11:**
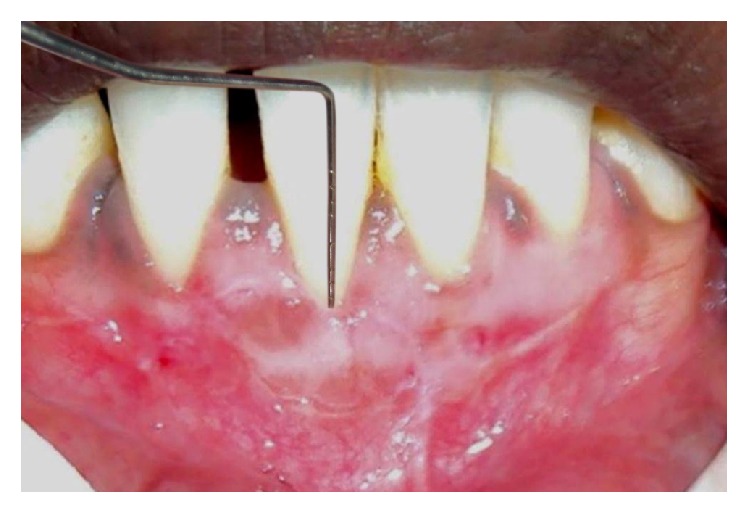
Postoperative view after 6 months showing 7 mm root coverage.

Results of 1st surgical procedure reported 5 mm of root coverage with 4 mm increase in attached gingiva (AG). There was further increase in AG of 4 mm with total root coverage of 7 mm as observed 6 months after stage two surgical procedure (Figure 11).

## 5. Discussion

There are multiple approaches documented in the literature for the treatment of gingival recession defects but there are not many dealing with the treatment of Miller class III recessions [8, 9]. The possible reason could be the unpredictability of success in treating these types of defect for complete root coverage (CRC). However, Blanes and Allen [10] stated that the achievement of CRC is possible in cases where the interproximal soft tissue integrity remained.

According to Lang and Loe [11], to attain CRC the primary objective was to widen the zone of AG in order to improve the periodontal health. Variety of techniques have been put forward, out of which the use of FGG, in a two-step procedure, is justified because of its predictability in treating extensive gingival recession in areas where adjacent donor tissue was nonexistent [12]. The present case report was treated by the same approach. This technique was first described by Bernimoulin et al. [13], where FGG was placed to increase the zone of keratinized gingiva (KG) and flap was coronally repositioned later for root coverage.

Studies by Maynard Jr. [14] stated that this approach was far superior because handling areas that involved thin adjacent gingiva made root coverage less predictable. Matter and Cimasoni [15] evaluated the same and discovered 65% root coverage on a predictable basis. In this case report postoperative results showed significant decrease of recession depth from 8 mm to 5 mm in first 3 months after the placement of FGG. There was 62.5% of root coverage and increase in zone of AG from 1 mm to 5 mm suggesting that FGG could be a predictable approach for the same. Wennström [16] observed that the average percentage of root coverage was nearly 72% in the FGG studies. Other studies have shown much better percentage of root coverage ranging from 80.3% to 100% [17–19].

The time interval between both the surgical procedures of 3 months provided good healing of the grafted site. The second stage root coverage performed by placing BioMend membrane showed 87.5% of root coverage. Other authors have quoted similar results for root coverage between 51% and 85% [20]. Additionally, increases in the amount of keratinized tissue after the GTR procedure have been found in the other studies [21–23] which explain further gain of AG by 3 mm in this report.

Pini Prato et al. [24] have shown that the GTR procedure is of greater efficacy in cases with severe recession. Collagen membranes have the capacity to partially prevent epithelial apical migration and to support new connective tissue attachment formation when used on denuded root surface [25]. Among the generations of resorbable membranes the predictability of BioMend is excellent [26].

The localized gingival recession treated using CAF and GTR membrane showed 100% coverage compared to CAF alone, showing the efficacy of this type of treatment [27]. Histometrically also, BioMend GTR along with CAF have shown a statistically significant gain of new attachment compared to CAF alone at 16 weeks [28].

The ultimate goal of periodontal therapy is the complete regeneration of the periodontal supporting tissues. The procedure holds promise for the successful management of complex marginal tissue recessions, although further studies are warranted.

## 6. Summary

The results obtained in this case suggest the following:

- The two-stage surgical procedure is highly predictable for root coverage in the case of deep recession and lack of attached gingiva in the mandibular anterior region.
- Use of GTR membrane (BioMend) has certain advantages such as (a) biocompatibility, (b) having no inflammatory reaction, (c) being easy to handle, cut, contour, and adapt, (d) maintaining desired shape and configuration, and (e) being easily secured in place and being completely absorbed by the host tissues.
- There is a need for long term trials to prove the efficacy of this technique.
